# Evaluation and Assessment of the Colour Stability of Modified Polymethyl Methacrylate Denture Base Materials and Polyether Ether Ketone in a Cast Partial Denture Framework: An In-Vitro Study

**DOI:** 10.7759/cureus.48189

**Published:** 2023-11-02

**Authors:** Joshua N, Nabeel Ahmed, Rekha Rattan

**Affiliations:** 1 Department of Prosthodontics, Saveetha Dental College, Saveetha Institute of Medical and Technical Sciences (SIMATS) Saveetha University, Chennai, IND

**Keywords:** heat cure acrylic resin, heat cure polymethyl methacrylate, dental colour measurement, peek removable partial denture, denture base material

## Abstract

Background

One of the frequent aesthetic issues patients confront is the loss of colour and lifeless appearance of the dentures of the base materials of their dentures after regular use. This leads to a lack of motivation to use the denture regularly. Due to the drawbacks of conventional PMMA, polyether ether ketone (PEEK) and newer polymethyl methacrylate (PMMA)-based materials have now started being used in cast partial denture frameworks due to their superior physical and biological properties. The lack of long-lasting colour is one of the main reasons for the repeat of dental prostheses. Hence, the need for the study is to help clinicians decide which would be the most suitable denture base material to be used based on colour stability.

Aim

To assess and compare the colour stability of PEEK, polyan, and biodentaplast denture base materials (DBMs) upon staining with distilled water, tea, coffee, and turmeric solutions after one day, seven days, and 30 days.

Methods

A total of 20 cuboidal specimens were constructed and immersed in distilled water, tea, coffee, and turmeric (five specimens of each material in each solution, a total of 60 specimens): Group 1: PEEK, Group 2: polyan, Group 3: biodentaplast. All specimens were subjected to colour measurements before exposure to beverage solutions, after 24 hours, on the seventh day and 30th day with a colour reflectance spectrophotometer with computer software. A one-way ANOVA test followed by post hoc Tukey’s honestly significant difference (HSD) was performed for comparison of colour stability between the DBMs, revealing a significant difference between PEEK and polyan and PEEK and biodentaplast. Polyan showed the highest delta E values, followed by biodentaplast and PEEK. A two-way ANOVA test, followed by Tukey’s HSD post hoc, was done to compare the staining ability of various staining solutions. Turmeric had the highest delta E values, followed by coffee, tea, and distilled water. Data were assessed using Statistical Product and Service Solutions (SPSS, version 23) (IBM SPSS Statistics for Windows, Armonk, NY) software.

Results

The highest mean delta E value at T1 was seen in biodentaplast immersed in turmeric (12.3900+/-0.442), and the least value at T1 was obtained for PEEK immersed in distilled water (0.4460+/-0.036). The highest mean delta E value at T2 was seen in polyan immersed in turmeric (13.0160+/-0.28962), and the least value at T2 was obtained for PEEK immersed in distilled water (0.5860+/-0.051). At T3, the highest mean delta E value was seen in polyan immersed in turmeric (16.8600+/-0.49845), and the least value at T3 was obtained for PEEK immersed in distilled water (0.700+/-0.037).

Conclusion

PEEK had the highest colour stability when compared with polyan and biodentaplast.

## Introduction

A cast partial denture is one of the recommended forms of removable prosthodontic treatment. However, with growing aesthetic concerns, it has recently been less opted for because of the evident colour change that results in an unappealing appearance over a period of prolonged use [1]. Any major variation in colour is an indication of aging or damaged material because colour stability is regarded as one of the most crucial clinical requirements for all dental materials [2]. The capacity of a material to retain its colour, despite environmental changes, is known as colour stability. Both inherent and external variables may result in colour changes in dental polymers. Intrinsic factors are those that are seen within the dental materials themselves, brought about by thermal and humidity alterations within the matrix as a result of physical and chemical conditions that play out during aging. Conversely, extrinsic factors comprise processes such as the absorption and adsorption of discolouring agents [3,4]. Various beverages such as coffee, tea, wine, and some food components in the Indian diet such as turmeric greatly augment the staining of denture base materials (DBMs) [5].

Given the aforementioned challenges, the dental industry continually searches for improved materials that can address the shortcomings of the currently available materials. Alongside polymethyl methacrylate (PMMA), the usage of thermoplastic resins and bio-functional prosthetic systems has greatly increased over the last decade. Despite having good functional, physical, and mechanical qualities, PMMA is known to be more prone to discolouration over time [1,3]. Of main focus in the recent past are thermoplastic resins such as Biodentaplast and Polyan. The use of thermoplastic resins in dental procedures is rapidly growing. The substance is only thermally plasticized; there is no chemical reaction.

Due to gradual improvements in chemical composition, it is today possible to create removable partial dentures with no metallic components using thermoplastic materials, giving rise to so-called 'metal-free removable partial dentures'. Polyether ether ketone (PEEK) is the newest material in dentistry, and it is reported to offer superior qualities compared to other materials [6]. PEEK is a semi-crystalline, high-temperature thermoplastic polymer having a high melting point. PEEK, which is radiolucent and has a flexural strength of 140-170 MPa, is extremely rigid and minimises magnetic resonance imaging artefacts [7,8]. The United States Food and Drug Administration (US FDA) Drug & Device Master files attest to its biocompatibility and bio-stability. It has a low specific weight and can be employed to create extremely lightweight prostheses that will increase patient comfort [9]. Additionally, the configuration promotes dental hygiene.

Despite adjustments being made to their composition to improve the strength and polishability, it is yet unknown whether these newer materials have the same discolouration tendencies as traditional PMMA or whether they can preserve their colour intra-orally. Coming to a consensus as to which permanent denture base material is most appropriate for prosthesis construction based on colour stability is of foremost importance. Hence, this study has been conducted to assess and compare the colour stability of PEEK, polyan, and biodentaplast DBMs upon staining with distilled water, tea, coffee, and turmeric solutions after one day, seven days, and 30 days.

## Materials and methods

This experimental in vitro study was carried out on 60 specimens of denture base materials. The sample size was estimated based on the study done by Banu et al. using G*Power 3.1.2 software (Heinrich-Heine-Universität Düsseldorf, Düsseldorf, Germany) with a power of 0.95 and p ≤ 0.05, and sample size derived was 20 specimens per group with the total sample size of 60 [1]. Before the start of the study, ethical clearance was obtained from the institutional ethics committee at Saveetha Institute of Medical and Technical Sciences (IHEC/SDC/PROSTHO-2102/23/245). All the specimens were randomly allocated to three groups of 20 specimens each using computer-generated randomization. They were numbered from one to 20 in each material group after the polishing protocol was completed. The computer software was then used to decide which sample would be placed in which media.

**Figure 1:**
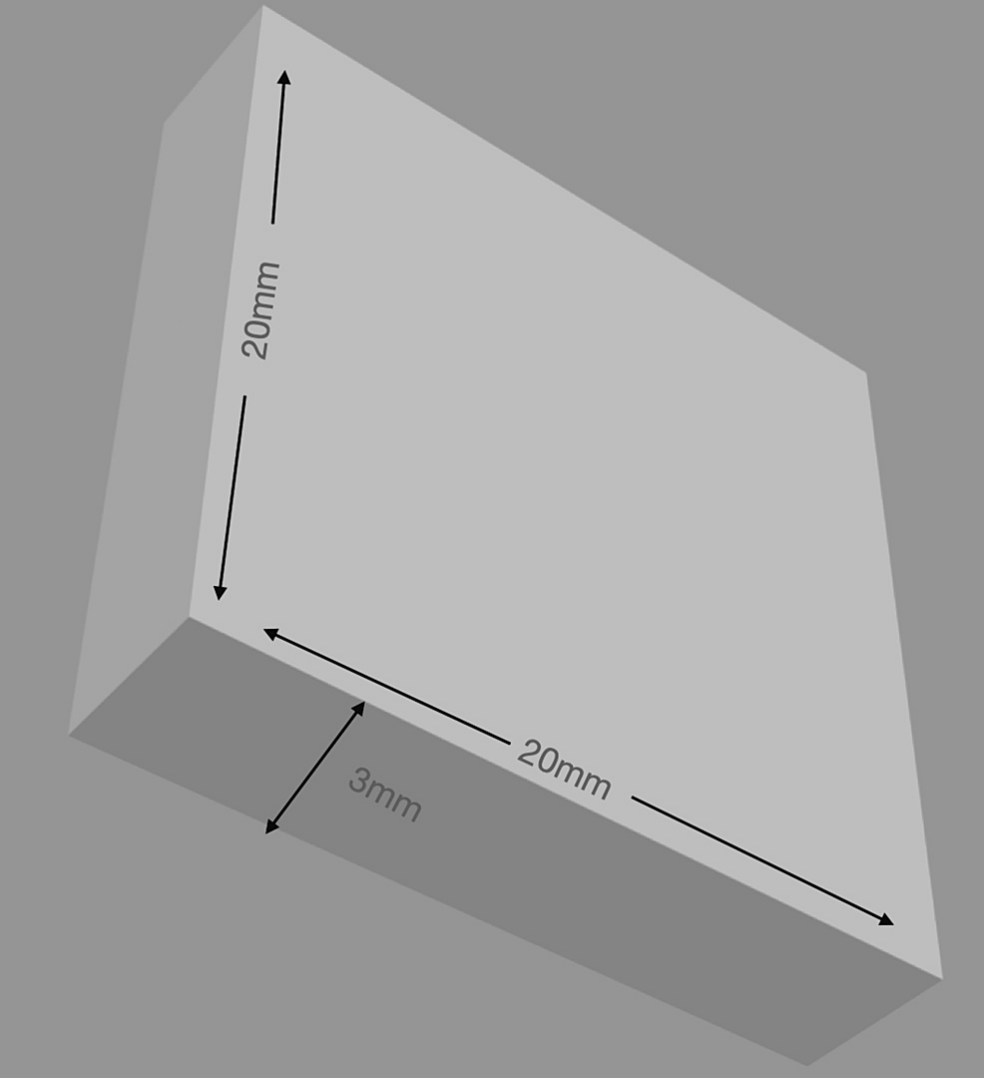
3D view of the wax pattern design

**Figure 2:**
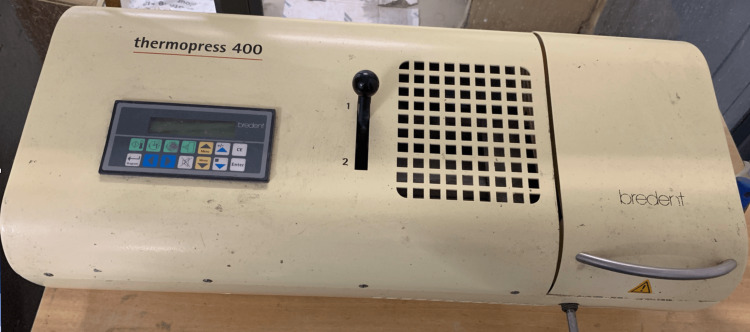
Thermopress 400

Three different types of DBMs were used to prepare the specimens, which were grouped as follows: Group 1: PEEK, Group 2: polyan, and Group 3: biodentaplast. Each cuboidal-shaped specimen had a size of 20 × 20 × 3 mm. Wax patterns were made based on the dimensions in Figure 1. They were then processed with polyan and biodentaplast using Thermopress 400 (Bredent Co., Senden, Germany) shown in Figure 2. PEEK specimens were prepared using PEEK blanks. The injection molding technique was followed.

Twenty specimens of each DBM were prepared. A tungsten carbide bur was used to remove extra resin from the specimens before finishing them with wet silicon carbide sheets (600-grit, 800-grit, 1,000-grit, and 1,200-grit). Only one surface was wet in order to simulate laboratory techniques, and it was polished with a cloth wheel and pumice. Wetting of only one surface was done since only one surface was polished in a real-life condition. This was the standard protocol that was followed for all the specimens. Specimens were subjected to 1,500 rounds of thermocycling to simulate the oral environment.

**Figure 3:**
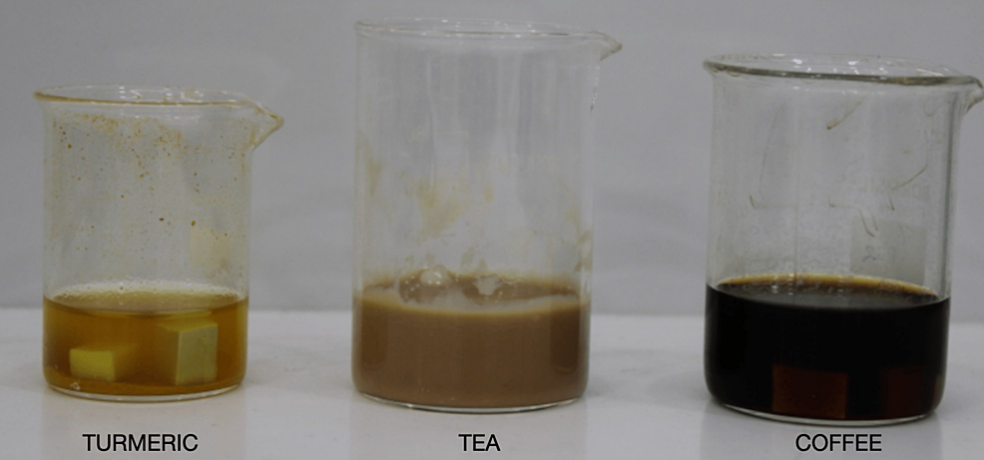
Preparation of tea, coffee, and turmeric solutions

The solutions for which color stability was checked in this study are distilled water, tea, coffee, and turmeric. Solutions of tea, coffee, and turmeric were concocted. Then, 8 g of coffee was added to 400 mL of boiling distilled water as per the manufacturer's instructions. One cup of coffee is approximately equal to 15-20 minutes and roughly around 3.2 cups. This was used as a standard value for the concoction. For tea, two readymade tea bags were dipped into 400 mL of water. The solution was made to cool for 15 minutes and then filtered using a gauze piece (Figure 3). Each solution, including distilled water, was divided into three parts so that five specimens of each DBM were immersed into their specific solutions. Each container with these specimens was placed in an incubator at a temperature of 37±1 °C. One time every three days, the solutions were refreshed. Refreshing is required to maintain the concentration of the solution for all three media. Additionally, solutions were agitated once a day to lessen particle precipitation in solutions. To reduce methodological variations and errors, the same operator prepared each solution.

**Figure 4:**
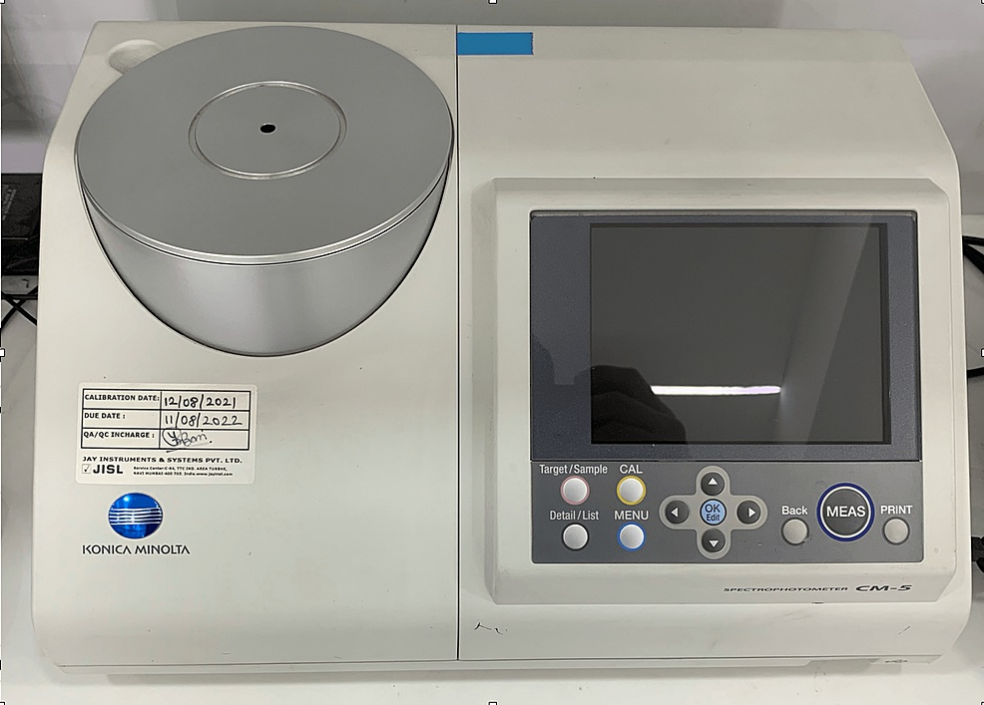
All specimens were subjected to colour measurements before exposure to beverage solutions, after 24 hours, on the seventh day and 30th day with a colour reflectance spectrophotometer

All specimens were subjected to colour measurements before exposure to beverage solutions, after the first day, on the seventh day, and on the 30th day, with a colour reflectance spectrophotometer with computer software (SpectraMagic NX, RM2002QC, Konica Minolta Corp., Ramsey, Japan) (Figure 4). Specifically, 24 hours of storage time simulated a month of drink consumption in the oral environment [10].

Before any measurement session, the colorimeter was calibrated in accordance with the manufacturer's instructions. Samples were placed on a standard white background plate for all measurements (No. 21633347, Konica Minolta Corp., Ramsey, NJ) with the background lighting switched on. Each specimen was placed in the spectrophotometer's viewport, and the measurements of each sample's L∗, a∗, and b∗ values were taken. After three times of measurements, the mean values for the L∗, a∗, and b∗ data were determined [11]. The measurements were carried out in the following timelines:

24 hours (T1): 24 Four hours after being submerged in the staining solutions

7 days (T2): 7 days after being submerged in the staining solutions

30 days (T3): 30 days after being submerged in the staining solutions

The samples were taken out of the staining solutions on the day of the assessment, dried with a three-way syringe, then cleaned with a dry cloth till there were no droplets of the media present on the specimen. This was carried out similarly for all the specimens, and then the first colour evaluation (T1) was carried out as previously described. The differences in the individual coordinate parameters between baseline (control) and after immersion in coloring solutions (T1) were calculated (∆E-delta E) from the following equation:

∆E = [(∆L (Lexperiment - L base))2 + (∆a (a experiment - a base))2 + (∆b (b experiment - b base))2 ] ½.

Here, ΔL* is the difference in lightness (L*) of the specimen following staining, Δa* is the difference in a* value (chroma on the red-green axis) of the specimen following staining, and Δb* is the difference in b* value (chroma on the yellow-blue axis) of the specimen following staining [12].

The higher the delta E value, the lower the colour stability. Data were organized and tested for normality using tests of normality (Kolmogorov-Smirnov and Shapiro-Wilk tests). Descriptive statistics were carried out to determine mean and standard deviation (SD) values. A one-way ANOVA test, followed by post hoc Tukey’s honestly significant difference (HSD), was performed for comparison between DBMs. A two-way ANOVA test, followed by post hoc Tukey’s HSD, was performed for comparison between different staining solutions. Statistics were analysed using Statistical Product and Service Solutions (SPSS, version 23) (IBM SPSS Statistics for Windows, Armonk, NY) software.

## Results

The highest mean 'delta E' value at T1 was seen in biodentaplast immersed in turmeric (12.3900+/-0.442), and the least value at T1 was obtained for PEEK immersed in distilled water (0.4460+/-0.036). Polyan displayed the highest delta E value at T2 immersed in turmeric (13.0160+/-0.28962), and the lowest value was of PEEK when immersed in distilled water (0.5860+/-0.051). Similarly at T3, the highest mean 'delta E' value was seen in polyan immersed in turmeric (16.8600+/-0.49845), and the lowest value at T3 was obtained for PEEK immersed in distilled water (0.700+/-0.037) (Table 1).

**Table 1:** Mean 'Delta E' values of the three denture base materials at different timelines when immersed in three different solutions DW - distilled water, T1 - after 24 hours, T2 - after 7 days, T3 - after 30 days

Material and time	Solution	N	Mean	Standard deviation
PEEK at T1	DW	5	0.4460	0.036
	Tea	5	3.1640	0.079
	Coffee	5	2.4920	0.172
	Turmeric	5	11.3360	0.255
PEEK at T2	DW	5	0.5860	0.051
	Tea	5	3.2380	0.061
	Coffee	5	2.7180	0.083
	Turmeric	5	11.3880	0.252
PEEK at T3	DW	5	0.700	0.037
	Tea	5	3.290	0.042
	Coffee	5	2.902	0.076
	Turmeric	5	15.552	199
Polyan at T1	DW	5	0.5940	0.10714
	Tea	5	4.6520	0.29269
	Coffee	5	3.2720	0.35864
	Turmeric	5	12.8800	0.36633
Polyan at T2	DW	5	0.7060	0.06986
	Tea	5	4.6940	0.28684
	Coffee	5	3.6020	0.27326
	Turmeric	5	13.0160	0.28962
Polyan at T3	DW	5	0.8040	0.09940
	Tea	5	4.7842	0.24395
	Coffee	5	3.8960	0.08112
	Turmeric	5	16.8600	0.49845
Biodentaplast at T1	DW	5	0.6020	0.08228
	Tea	5	4.6060	0.37407
	Coffee	5	3.5780	0.13700
	Turmeric	5	12.3900	0.44210
Biodentaplast at T2	DW	5	0.7260	0.09397
	Tea	5	4.6460	0.38175
	Coffee	5	3.8040	0.15534
	Turmeric	5	12.3900	0.44210
Biodentaplast at T3	DW	5	0.8060	0.02702
	Tea	5	4.7020	0.34040
	Coffee	5	3.8760	0.08933
	Turmeric	5	16.6220	0.40856

There was no significant difference observed between polyan and biodentaplast (p=0.150). The greatest difference in 'delta E' values was seen between PEEK and polyan. PEEK exhibited the lowest 'delta E' values, which show the highest colour stability, and polyan exhibited the highest 'delta E' values corresponding to the lowest colour stability in Table 2.

**Table 2:** Comparison of mean 'delta E' values between different denture base materials using post hoc Tukey’s HSD

Groups		Mean difference	Standard error	Significance
PEEK	Polyan	0.9957	0.044	0
	Biodentaplast	0.9113	0.044	0
Polyan	PEEK	0.9957	0.044	0
	Biodentaplast	0.0844	0.044	0.15
Biodentaplast	PEEK	0.9113	0.044	0
	Polyan	0.0844	0.044	0.15

A two-way ANOVA test was performed to assess the difference in staining between the four coloring solutions with respect to dental material and time. Significant differences were observed between the solutions (p<0.05). The 'delta E' values were significantly higher for turmeric followed by tea, followed by coffee indicating a higher staining ability for turmeric followed by tea, followed by coffee.

## Discussion

DBMs must be present in an environment with varying oral temperatures, salivary pH, and its constituents as well as contact with a variety of foods and beverages consumed at scalding temperatures making it susceptible to changes in their physical makeup and appearance brought on by the uptake of various contaminants [1].

In the current study, the colour stability of PEEK, polyan, and biodentaplast was examined under the influence of distilled water, tea, coffee, and turmeric. Visual inspection is a self-reported physiological and psychological process used to evaluate colour changes. Contrarily, when a colour change is determined using a spectrophotometer, it not only eliminates subjective interpretations but also enables the identification of minute colour changes [12,13]. A colour system with the name "Commission Internationale de l’Eclairage (CIE) L*a*b" is a widely used standard color scale. All the colours discernible to the human eye are included in the continuous colour scale L*a*b. Therefore, it is suitable for research on how the hue of dental materials changes perceivably [13,14].

A difference in colour with corresponding ΔE* values lesser than 3.3 is acceptable for clinical dentist practice. In the current study, the ΔE* values of all groups exceeded 3.3, except for distilled water [15]. In the current study, turmeric produced the highest staining, which is in agreement with a study done by Singh et al. [16]. The main colour component present in turmeric, curcumin, is what causes the staining. The active component of turmeric, curcumin, has a vivid yellow colour and is frequently used as a food colouring agent. Moreover, in this study, coffee stained more than tea. Tea and coffee both are known to cause varying changes in the levels of the colour stability. The process of adsorption and absorption are both known to cause the staining of DBMs. Coffee's tannin, caffeine, and water-soluble polyphenols are less polar colorants that are more compatible with polymer matrices; therefore, they may have deeply imbibed into the substance [10]. The high molecular weight of water-soluble substances plays a role in reduced colour stability. This outcome was consistent with several other types of research showing that specimens of heat-polymerised resin DBMs were more discoloured by coffee solution than by tea solution [17,18].

In the present study, colour stability significantly decreased with time. DBMs must be present in an environment with varying oral temperatures, salivary pH, and its constituents, as well as contact with a variety of foods and beverages consumed at a variety of temperatures, making it susceptible to changes in their physical makeup and appearance as a result of the consumption of these substances. Although the specimens used comprise modified acrylic resins, its internal structure is still made up of organic compounds. These compounds are likely to lose some of their translucency and colour stability as a result of colourants adhering to the surface pellicle layer that forms on DBMs when they come into contact with various chemicals found in food and drink products. This is in accordance with a study done by Zuo et al. who established that the discoloration was time-dependent and increased with longer immersion times [18].

In a study conducted by Banu et al. comparing thermoplastic resin to traditional PMMA and high-impact PMMA at both the 24-hour mark and after immersion in the denture cleaner, thermoplastic resin demonstrated improved colour stability [1]. In the present study, PEEK showed the highest colour stability. The reason for this is due to the molecular structure of PEEK, which permits lesser absorption of the colourants into the matrix, hence increasing the stability of the materials. Heimer et al. analysed the effect on the colour stability and stain removal function of various cleaning methods on PEEK and polyoxymethylene (POM) using different media for seven days and reported that PEEK had the highest colour stability, lowest water absorption, and solubility in comparison with PMMA and composite resin [19]. The authors added that POM's increased discolouration was most likely brought on by its surface's coarser texture than PEEK's after polishing. Additionally, there is a possibility that POM discolours more than PEEK because it absorbs and diffuses water more readily than PEEK, which has 20% ceramic fillers; however, more research is needed for the same. Surface free energy and surface roughness have a considerable impact on colour stability [20]. Numerous investigations revealed a link between denture resin discolouration and high surface roughness, which can be substantiated by the large surface area with the variety of preference sites for agglomerating colourants [20,21].

In prosthodontics, colour stability in DBMs is crucial. Because they are so noticeable in the mouth cavity, dentures require both aesthetic and practical attributes. Polymethyl methacrylate is a polymer that can change colour over time as a result of contact with environmental conditions, oral hygiene products, and food stains. To preserve a natural appearance and avoid deterioration that could be socially awkward for wearers, colour stability is essential.

Patients' self-esteem and confidence are maintained by denture colour stability, allowing them to comfortably interact in social and professional contexts. Additionally, it lessens the need for regular replacements, saving both patients and dental professionals time and money. Because of this, selecting DBMs with colour stability is essential for prosthodontic procedures that aim for long-lasting, aesthetically acceptable outcomes.

The present findings are only a promising starting point for further research due to the limitations of this study, which include the absence of human saliva and denture biofilm that may affect the results of colour change and cause inaccurate prediction of the performance of the materials being tested. The use of only certain media could be seen as another limitation of this study. The use of different media, such as juices containing citric acids, aerated beverages, and milk, might provide us with more findings. Additionally, the use of a straightforward cuboid-shaped specimen, which does not accurately represent the shape of a denture framework, could be seen as a possible limitation. The polymerisation method, several kinds of denture bases, and the incorporation of nanoparticles should be the focus of future research. Denture care and the maintenance should also be considered an important factor in the long-term use of a denture. Research should also be conducted in this spectrum with similar materials used.

## Conclusions

Within the limitations of the study, it can be concluded that PEEK has a higher color stability in comparison with modified PMMA materials. Hence, it can be regarded as a promising potential alternative to metals. Long-term studies should look into how the diverse oral environment affects the aging behavior of denture base materials.

## Appendices

Significance testing in colour stability between the immersion groups using a two-way ANOVA test is given in Table 3.

**Table 3:** Significance testing in colour stability between the immersion groups using a two-way ANOVA test

Evaluation period	Groups	Immersion solution				Sum of Squares	Mean Square	Sig.
24 hours	PEEK	DW	Coffee	Tea	Turmeric	344.519	114.840	0.000
	POLY	DW	Coffee	Tea	Turmeric	420.629	140.210	0.000
	BIO	DW	Coffee	Tea	Turmeric	378.930	126.310	0.000
7th day	PEEK	DW	Coffee	Tea	Turmeric	337.654	112.551	0.000
	POLY	DW	Coffee	Tea	Turmeric	418.623	139.541	0.000
	BIO	DW	Coffee	Tea	Turmeric	369.109	123.036	0.000
30th day	PEEK	DW	Coffee	Tea	Turmeric	678.336	226.112	0.000
	POLY	DW	Coffee	Tea	Turmeric	747.346	249.115	0.000
	BIO	DW	Coffee	Tea	Turmeric	724.973	241.658	0.000

Tukey’s honestly different significant post hoc was performed for pair-wise comparisons at 24 hours, seven days, and 30 days, as given in Table 4.

**Table 4:** Tukey’s honestly different significant post hoc was performed for pair-wise comparisons at 24 hours, seven days, and 30 days

Multiple Comparisons					
Tukey HSD				
Dependent Variable			Mean Difference	Std. Error
PEEK at 24 hours	DW	Tea	-2.71800	0.10127
		Coffee	-2.04600	0.10127
		Turmeric	-10.89000	0.10127
	Tea	DW	2.71800	0.10127
		Coffee	.67200	0.10127
		Turmeric	-8.17200	0.10127
	Coffee	DW	2.04600	0.10127
		Tea	-.67200	0.10127
		Turmeric	-8.84400	0.10127
	Turmeric	DW	10.89000	0.10127
		Tea	8.17200	0.10127
		Coffee	8.84400	0.10127
Polyan at 24 hours	DW	Tea	-4.05800	0.18973
		Coffee	-2.67800	0.18973
		Turmeric	-12.28600	0.18973
	Tea	DW	4.05800	0.18973
		Coffee	1.38000	0.18973
		Turmeric	-8.22800	0.18973
	Coffee	DW	2.67800	0.18973
		Tea	-1.38000	0.18973
		Turmeric	-9.60800	0.18973
	Turmeric	DW	12.28600	0.18973
		Tea	8.22800	0.18973
		Coffee	9.60800	0.18973
Biodentaplast at 24 hours	DW	Tea	-4.00400	0.18998
		Coffee	-2.97600	0.18998
		Turmeric	-11.78800	0.18998
	Tea	DW	4.00400	0.18998
		Coffee	1.02800	0.18998
		Turmeric	-7.78400	0.18998
	Coffee	DW	2.97600	0.18998
		Tea	-1.02800	0.18998
		Turmeric	-8.81200	0.18998
	Turmeric	DW	11.78800	0.18998
		Tea	7.78400	0.18998
		Coffee	8.81200	0.18998
PEEK at 7 days	DW	Tea	-2.65200	0.08777
		Coffee	-2.13200	0.08777
		Turmeric	-10.80200	0.08777
	Tea	DW	2.65200	0.08777
		Coffee	.52000	0.08777
		Turmeric	-8.15000	0.08777
	Coffee	DW	2.13200	0.08777
		Tea	-.52000	0.08777
		Turmeric	-8.67000	0.08777
	Turmeric	DW	10.80200	0.08777
		Tea	8.15000	0.08777
		Coffee	8.67000	0.08777
Polyan at 7 days	DW	Tea	-3.98800	0.15675
		Coffee	-2.89600	0.15675
		Turmeric	-12.31000	0.15675
	Tea	DW	3.98800	0.15675
		Coffee	1.09200	0.15675
		Turmeric	-8.32200	0.15675
	Coffee	DW	2.89600	0.15675
		Tea	-1.09200	0.15675
		Turmeric	-9.41400	0.15675
	Turmeric	DW	12.31000	0.15675
		Tea	8.32200	0.15675
		Coffee	9.41400	0.15675
Biodentaplast at 7 days	DW	Tea	-3.92000	0.19343
		Coffee	-3.07800	0.19343
		Turmeric	-11.66400	0.19343
	Tea	DW	3.92000	0.19343
		Coffee	.84200	0.19343
		Turmeric	-7.74400	0.19343
	Coffee	DW	3.07800	0.19343
		Tea	-.84200	0.19343
		Turmeric	-8.58600	0.19343
	Turmeric	DW	11.66400	0.19343
		Tea	7.74400	0.19343
		Coffee	8.58600	0.19343
PEEK at 30 days	Distilled Water	Tea	-2.59000	0.06978
		Coffee	-2.20200	0.06978
		Turmeric	-14.85200	0.06978
	Tea	Distilled Water	2.59000	0.06978
		Coffee	0.38800	0.06978
		Turmeric	-12.26200	0.06978
	Coffee	Distilled Water	2.20200	0.06978
		Tea	-0.38800	0.06978
		Turmeric	-12.65000	0.06978
	Turmeric	Distilled Water	14.85200	0.06978
		Tea	12.26200	0.06978
		Coffee	12.65000	0.06978
Polyan at 30 days	Distilled Water	Tea	-3.98020	0.18012
		Coffee	-3.09200	0.18012
		Turmeric	-16.05600	0.18012
	Tea	Distilled Water	3.98020	0.18012
		Coffee	0.88820	0.18012
		Turmeric	-12.07580	0.18012
	Coffee	Distilled Water	3.09200	0.18012
		Tea	-0.88820	0.18012
		Turmeric	-12.96400	0.18012
	Turmeric	Distilled Water	16.05600	0.18012
		Tea	12.07580	0.18012
		Coffee	12.96400	0.18012
Biodentaplast at 30 days	Distilled Water	Tea	-3.89600	0.17073
		Coffee	-3.07000	0.17073
		Turmeric	-15.81600	0.17073
	Tea	Distilled Water	3.89600	0.17073
		Coffee	0.82600	0.17073
		Turmeric	-11.92000	0.17073
	Coffee	Distilled Water	3.07000	0.17073
		Tea	-0.82600	0.17073
		Turmeric	-12.74600	0.17073
	Turmeric	Distilled Water	15.81600	0.17073
		Tea	11.92000	0.17073
		Coffee	12.74600	0.17073
